# *Tiller Angle Control 1* Is Essential for the Dynamic Changes in Plant Architecture in Rice

**DOI:** 10.3390/ijms23094997

**Published:** 2022-04-30

**Authors:** Hong Wang, Ranran Tu, Lianping Sun, Dongfei Wang, Zheyan Ruan, Yue Zhang, Zequn Peng, Xingpeng Zhou, Junlin Fu, Qunen Liu, Weixun Wu, Xiaodeng Zhan, Xihong Shen, Yingxin Zhang, Liyong Cao, Shihua Cheng

**Affiliations:** 1 Key Laboratory for Zhejiang Super Rice Research, State Key Laboratory of Rice Biology, China National Center for Rice Improvement, China National Rice Research Institute, Hangzhou 311401, China; wjiyinh@126.com (H.W.); 18883948050@163.com (R.T.); sunlianping@caas.cn (L.S.); w553055@126.com (D.W.); skyhesea@163.com (Z.R.); zhangyuerice@163.com (Y.Z.); 13720149899@163.com (Z.P.); zhouxingpeng@126.com (X.Z.); fujunlin@caas.cn (J.F.); liuqunen202@163.com (Q.L.); wuweixun@caas.cn (W.W.); zhanxiaodeng@caas.cn (X.Z.); xihongshen@126.com (X.S.); zhangyingxin@caas.cn (Y.Z.); 2 Rice Research Institute, Key Laboratory of Application and Safety Control of Genetically Modified Crops, Academy of Agricultural Sciences, Southwest University, Chongqing 400715, China

**Keywords:** rice (*Oryza sativa* L.), plant architecture, dynamic changes, gene mapping, *TAC1*

## Abstract

Plant architecture is dynamic as plants develop. Although many genes associated with specific plant architecture components have been identified in rice, genes related to underlying dynamic changes in plant architecture remain largely unknown. Here, we identified two highly similar recombinant inbred lines (RILs) with different plant architecture: RIL-Dynamic (D) and RIL-Compact (C). The dynamic plant architecture of RIL-D is characterized by ‘loose^tiller angle^ (tillering stage)–compact (heading stage)–loose^curved stem^ (maturing stage)’ under natural long-day (NLD) conditions, and ‘loose^tiller angle^ (tillering and heading stages)–loose^tiller angle and curved stem^ (maturing stage)’ under natural short-day (NSD) conditions, while RIL-C exhibits a compact plant architecture both under NLD and NSD conditions throughout growth. The candidate locus was mapped to the chromosome 9 tail via the rice 8K chip assay and map-based cloning. Sequencing, complementary tests, and gene knockout tests demonstrated that *Tiller Angle Control 1* (*TAC1*) is responsible for dynamic plant architecture in RIL-D. Moreover, *TAC1* positively regulates loose plant architecture, and high *TAC1* expression cannot influence the expression of tested tiller-angle-related genes. Our results reveal that *TAC1* is necessary for the dynamic changes in plant architecture, which can guide improvements in plant architecture during the modern super rice breeding.

## 1. Introduction

Plant architecture typically refers to morphological characteristics associated with the three-dimensional organization of the plant body, including plant height, tiller number or angle, and inflorescence structure, which represents the major agronomic traits in the field [1]. Ideal plant architecture is one of the physiological characteristics for high yields, and improving plant architecture plays a vital role in the breeding of modern crops. A better understanding of the molecular basis underlying plant architecture will contribute to improvements in plant architecture.

Plant architecture determines planting density and substantially affects stress resistance, lodging, and light capture/photosynthetic efficiency [2], and planting density largely depends on the branch angle in plants [3], such as the tiller angle in rice [4]. *Tiller Angle Control 1* (*TAC1*) was initially identified for controlling tiller angle in an *indica* variety IR24; a mutation from ‘AGGA’ to ‘GGGA’ (functional nucleotide polymorphism, FNP) occurring at the splicing site of the fourth intron in the 3′-untranslated region (3′-UTR) decreases the expression of the *tac1* allele, producing a compact plant architecture with a tiller angle close to zero. Overexpressing *TAC1* in Nipponbare (NPB) leads to a larger tiller angle, and repressing *TAC1* expression through RNA interference results in a more compact plant architecture. These results demonstrate the wide application of the *tac1* allele in *japonica* cultivars during rice domestication [5,6]. Thereafter, *TAC1* was identified as a key regulator for enlarging the branch angle/leaf angle in other plant species, such as maize, peach, Arabidopsis, and plum, and it was defined as a member of the IGT/LAZY gene family [7,8,9]. WEEP, a sterile alpha motif protein, is involved in gravitropic responses and controls weeping tree architecture in peach and plum species [9]; it was suggested to be a factor downstream of the TAC1 pathway [10]. *LAZY1* (*LA1*), which is another member of the IGT/LAZY gene family, negatively regulates polar auxin transport (PAT) to determine rice shoot gravitropism and tiller angle [11], and Brevis Radix-Like 4 (OsBRXL4) can interact with LA1 at the plasma membrane; this interaction determines the nuclear localization of the LA1 protein, thus regulating tiller angle in rice [12]. In addition, *LAZY1* and the other homologs, *LAZY2* and *LAZY4*, were reported to perceive gravistimulation to influence the local auxin gradient in plants [10,13,14]. Maize *ZmLAZY1* regulates gravitropic responses, but not the leaf angle [13], while another ortholog, *ZmCLA4*, modulates the leaf angle by influencing the cell shape and number at the leaf axil [15]. Notably, although *LAZY1* and *TAC1* show opposing influences on branch angle in Arabidopsis, the connections between these two genes are complex, and they are not the direct negative regulators of each other [16]. Furthermore, several regulators upstream or downstream of *LA1* have been identified in rice. HEAT STRESS TRANSCRIPTION FACTOR 2D (HSFA2D) acts as an upstream regulator to positively regulate *LA1* expression [17], and two class II homeodomain–leucine zipper (HD-ZIP II) proteins, OsHOX1 and OsHOX28, act upstream of *OsHSFA2D,* and can bind to the promoter of *OsHSFA2D* to suppress its expression [18]. *LA1* can promote auxin accumulation in lower parts of the plant upon gravistimulation, leading to the asymmetric expression of the downstream auxin regulators *WUSCHEL-RELATED HOMEOBOX 6* (*WOX6*) and *WOX11*. A double mutant of these two genes results in a larger tiller angle [17]. *PROSTRATE GROWTH 1* (*PRGO1*), encoding a Cys_2_–His_2_ zinc finger protein, controls prostrate growth in wild rice, and *PRGO1* deficiency leads to erect growth in domesticated rice cultivars [19,20]. *TILLER INCLINED GROWTH 1* (*TIG1*), encodes a TCP transcriptional activator, promotes cell elongation, and increases the tiller angle in wild rice; variations in the promoter of the *tig1* allele from *indica* lead to decreased expression, and reduced cell length and tiller angle, resulting in erect growth of the tiller during rice domestication [21]. *LAZY2* (*LA2*)/*Large Tiller Angle 1* (*LTA1*) encodes a chloroplastic protein that interacts with a starch biosynthetic enzyme, *Oryza sativa* plastidic phosphoglucomutase (OspPGM), to regulate starch biosynthesis in gravity-sensing cells, thus controlling shoot gravitropism and tiller angle [22,23]. *TAC4* encodes a plant-specific, highly conserved nuclear protein, which affects the indole acetic acid (IAA) content and auxin distribution to regulate shoot gravitropism and tiller angle [24].

Additionally, many genes or related regulatory factors can simultaneously control several components of plant architecture to modulate overall plant architecture in rice. *Ideal Plant Architecture 1* (*IPA1*) encodes squamosa promoter binding protein-like 14 (OsSPL14), which negatively regulates tiller development (shoot branching) in the vegetative stage, and promotes panicle branching in the reproductive stage, increasing grain yield [25,26,27]. Overexpression of the auxin efflux transporter gene *OsPIN2* increases tiller number and tiller angle, and decreases plant height [28]. *Loose Plant Architecture 1* (*LPA1*) encodes a plant-specific INDETERMINATE DOMAIN protein and a functional ortholog of the *AtIDD15*/*SHOOT GRAVITROPISM5* (*SGR5*) gene in Arabidopsis, and regulates the sedimentation rate of amyloplasts to affect gravity perception or signal transduction in coleoptile gravitropism. Additionally, it can suppress the auxin signaling that interacts with C-22-hydroxylated and 6-deoxo BRs, thus simultaneously controlling tiller angle and leaf angle [29,30]. The *PLANT ARCHITECTURE AND YIELD 1* (*PAY1*) mutant displays a smaller tiller angle, higher plant height, lower tiller number, larger panicles, thicker stems, and enhanced grain yield in comparison with the control line YIL55. Further studies have demonstrated that *PAY1* affects PAT activity and alters endogenous IAA distribution to improve rice plant architecture [31]. A transcription factor *OsbZIP49* from the bZIP family of the TGA class influences local auxin homeostasis to modulate tiller angle and plant height [32].

Most studies have focused on the molecular mechanisms underlying the specific components of plant architecture, such as plant height, tiller angle or number, and panicle branching or development. Notably, plant architecture is related to growth and development, and changes in response to internal and external factors, making it dynamic as plants develop [33,34]. However, the underlying molecular basis of plant architecture remains largely unclear, likely due to the complexity of such dynamic changes during plant development. In this study, we identified and characterized two highly similar recombinant inbred lines (RILs) with different plant architecture: the RIL-D (Dynamic) is characterized by ‘loose^tiller angle^ (tillering stage)–compact (heading stage)–loose^curved stem^ (maturing stage)’ under natural long-day (NLD) conditions, and ‘loose^tiller angle^ (tillering and heading stages)–loose^tiller angle and curved stem^ (maturing stage)’ under natural short-day (NSD) conditions, while RIL-C (Compact) is characterized by a compact plant architecture throughout their growth. Gene mapping, sequencing, and transgenic tests all demonstrated that *TAC1* was the target gene responsible for the dynamic plant architecture. Our results demonstrate that the *TAC1* gene modulates the dynamic plant architecture in rice, indicating a potential target for improving plant architecture during the breeding of modern super rice.

## 2. Results

### 2.1. RIL-D Shows the Dynamic Plant Architecture under Natural Long-Day Conditions

To identify the gene responsible for regulating dynamic plant architecture, we constructed a recombinant inbred line (RIL) population of F_6:7_ by crossing the *indica* variety (ZH8015) with a *japonica* variety (02428), and identified two highly similar RILs with different plant architecture, named RIL-D (the RIL with dynamic plant architecture) and RIL-C (the RIL with compact plant architecture).

**Figure 1:**
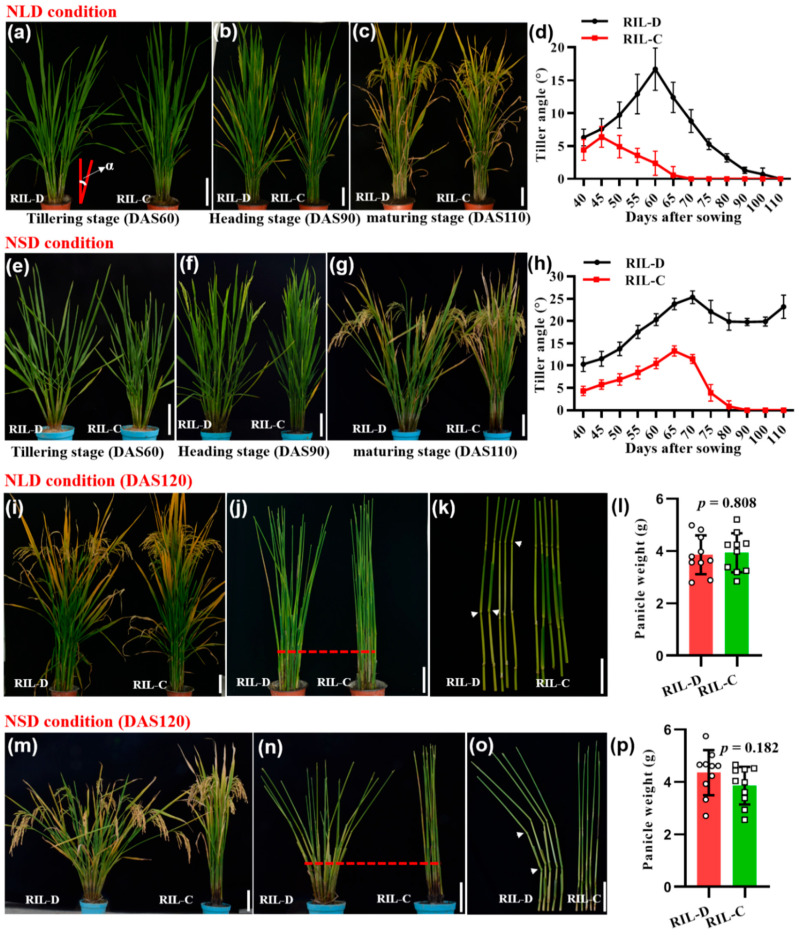
Phenotypic analysis of RIL-D and RIL-C from DAS40 to DAS110 under natural long-day (NLD) and short-day (NSD) conditions. (**a**–**c**) RIL-D and RIL-C plants under NLD conditions at the tillering stage (**a**), heading stage (**b**), and maturing stage (**c**). (**d**) The dynamic tiller angle of RIL-D and RIL-C under NLD conditions. (**e**–**g**) RIL-D and RIL-C plants under NSD conditions at the tillering stage (**e**), heading stage (**f**), and maturing stage (**g**). (**h**) The dynamic tiller angle of RIL-D and RIL-C under NSD conditions. (**i**) RIL-D and RIL-C plants under NLD conditions. (**j**,**k**) Stems of RIL-D and RIL-C under NLD conditions. (**l**) Comparison of the panicle weight between RIL-D and RIL-C under NLD conditions. (**m**) RIL-D and RIL-C plants under NSD conditions. (**n,o**) Stems of RIL-D and RIL-C under NSD conditions. (**p**) Comparison of the panicle weight between RIL-D and RIL-C under NSD conditions. The upper and lower parts of red dotted lines indicate curved stems and tiller angle, respectively, and white arrows indicate the curved site (node) of the stem. All data shown represent mean ± SD (standard deviation; *n* = 10). Two-tail Student’s *t*-tests were used for statistical analysis. Bar = 10 cm.

Under natural long-day (NLD) conditions (the summer growing season in Fuyang (119°95′ E, 30°05′ N), Zhejiang province, China), RIL-D displayed a loose plant architecture characterized by a tiller angle that gradually increased until 60 days after sowing (DAS60, tillering stage), and reached a maximum of ~16.7° at DAS60 (Figure 1a,d). In contrast, the average tiller angle of RIL-C increased until DAS45, reached a maximum of ~6.4°, after which it steadily decreased until DAS60, where it was close to zero (Figure 1a,d). After DAS60, the RIL-D tiller angle sharply decreased, and by the heading stage (DAS90) its architecture was compact with no obvious difference from RIL-C plants (Figure 1b–d). At the late-maturing stage (DAS120), RIL-D showed a loose plant architecture due to greater curvature in the stem, although its tiller angle remained close to zero (Figure 1i–k), and there was no significant difference in the panicle weight of RIL-D and RIL-C (Figure 1l). This suggests that the curved stem of RIL-D was unrelated to differences in panicle weight. These observations indicate that RIL-D had an overall dynamic plant architecture characterized by ‘loose^tiller angle^ (tillering stage)–compact (heading stage)–loose^curved stem^ (maturing stage)’, while RIL-C had a relatively compact plant architecture throughout the growth period under NLD conditions.

Under natural short-day (NSD) conditions (the winter growing season in Lingshui (110°02′ E, 18°48′ N), Hainan province, China), RIL-D displayed a loose plant architecture featuring a large, continuously increasing tiller angle (~25.5° maximum) until DAS70 (Figure 1e–h). Although the tiller angle of RIL-D decreased after DAS70, it stabilized at ~20.0° from DAS80 to DAS100, and increased after DAS100 (Figure 1h). In contrast to this loose, dynamic architecture, RIL-C showed a relatively compact plant architecture (Figure 1e–h and Figure 1m–o). In addition, RIL-D also exhibited a curved stem, with no significant difference in panicle weight between RIL-D and RIL-C at the late maturation stage (DAS120; Figure 1m–p). Moreover, the RIL-D and RIL-C plants grown in the winter season (the NSD conditions of the Fuyang greenhouse) had a plant architecture similar to that of their respective counterparts grown under the NSD conditions (Figure S1a–d). Taken together, RIL-D showed a loose plant architecture characterized by ‘loose^tiller angle^ (tillering and heading stages)–loose^tiller^ ^angle and curved stem^ (maturing stage)’, while RIL-C had a relatively compact plant architecture throughout their growth stages under NSD conditions.

### 2.2. Genetic Analysis and Fine Mapping of the Candidate Gene

To investigate whether the RIL-D phenotype is controlled by a single gene, we phenotypically scored 500 plants at maturing stage (DAS110) for each line of three segregated lines (HNPF-270/273/274; Figure S2) in the F_6:7_ RIL population under NSD conditions. The segregation models for loose-to-compact plants fit a 3:1 Mendelian ratio (Table S1), suggesting that the phenotype of RIL-D was controlled by a single dominant locus.

To map the candidate gene, we initially selected 30 plants with loose plant architecture and 64 plants with compact plant architecture from different F_6:7_ lines, which were genotyped using the rice 8k chip (Table S2 and Figure S3). This produced 25 heterozygous or cross-over regions (Figure S4 and Table S3). The general linear model (GLM) and mixed linear model (MLM) were then used to detect the candidate region using the Tassel 5.0 software (Ithaca, New York, NY, United States), and the results show that the candidate region was located at the tail of chromosome 9, which was used as the preliminary linkage interval within 2371.5 Kb between the SNP markers AX-115869042 and AX-95959392 (Figures S4 and S5).

**Figure 2:**
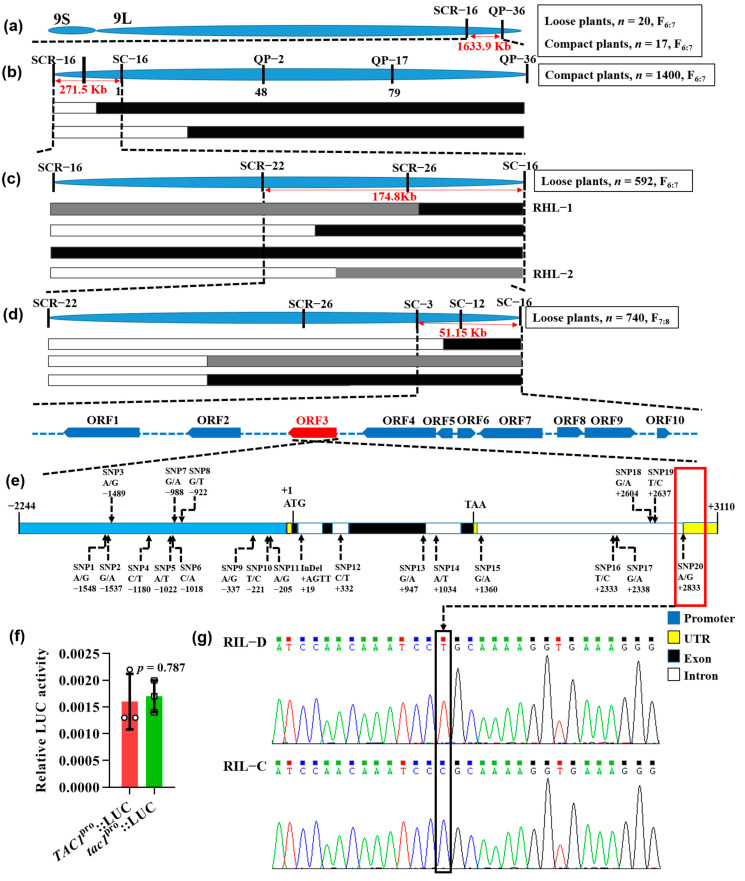
Map-based cloning, sequencing, and promoter activity assay of *TAC1*. (**a**) The preliminary linkage interval was mapped to a 1633.9 Kb interval between InDel markers SCR-16 and QP-36 at the tail of chromosome 9 using 20 loose plants and 17 compact plants from the F_6:7_ RIL population at DAS110 under natural short-day (NSD) conditions. (**b**) The candidate locus was narrowed down to a 271.5 Kb region flanked by SCR-16 and SC-16 using 1400 compact plants from the F_6:7_ RIL population. (**c**) The mapping interval was further narrowed down to a 174.8 Kb region between SCR-22 and SC-16 using 592 loose plants from the F_6:7_ RIL population, while two heterozygous plants were selected for generating RHL-1 and RHL-2. (**d**) The candidate region containing 10 ORFs was finally mapped to a 51.15 Kb interval between SC-3 and SC-16 using 740 loose plants from RHL-2 at DAS60 under natural long-day (NLD) conditions. (**e**) Sequencing analysis of the 5354 bp *TAC1* genomic sequence. (**f**) The *TAC1* (*tac1*) promoter activity assays with the Dual-Luciferase^®^ Reporter Assay System. Data are displayed mean ± SD (*n* = 3). Two-tail Student’s *t*-test was used for statistical analysis. (**g**) DNA sequencing chromatograms of the splicing sites of 3′-untranslated region of *TAC1* between RIL-D and RIL-C.

To finely map the candidate gene, 16 polymorphic insertion/deletion (InDel) markers evenly distributed on the preliminary linkage interval were developed to genotype 20 loose plants and 17 compact plants from F_6:7_ at DAS110 under NSD conditions. Therefore, the preliminary linkage interval was mapped to a region within 1633.9 Kb between the InDel markers SCR-16 and QP-36 (Figure 2a). To narrow down the interval, 1400 compact plants from F_6:7_ were genotyped, and then the interval was mapped to the region within 271.5 Kb flanked by the InDel markers between SCR-16 and SC-16 (Figure 2b). Two new polymorphic InDel markers were developed in the 271.5 Kb region, and a total of four polymorphic InDel markers (including SCR-16 and SC-16) were used to genotype 592 loose plants from F_6:7_. This enabled the candidate region to be narrowed down to a 174.8 Kb interval between SCR-22 and SC-16 (Figure 2c). Unfortunately, we could not obtain the expected interval using all the sampled individuals, but we did obtain two heterozygous plants genotyped by SCR-26 (Figure 2c). We then harvested all the seeds of these two heterozygous plants and generated two residual heterozygous lines (RHLs), RHL-1 and RHL-2, under natural long-day (NLD) conditions. These included segregated individuals only found in RHL-2. Subsequently, 740 loose plants in RHL-2 were sampled to map the candidate region with five polymorphic InDel markers (two newly developed markers) to a 51.15 Kb region, which contained 10 open reading frames (ORFs; Figure 2d) according to the Rice Annotation Project (RAP) database (https://rapdb.dna.affrc.go.jp/ (accessed on 25 February 2019)).

Of the 10 ORFs in the candidate region, ORF3 is the *TAC1* gene previously identified as a major QTL for rice tiller angle [5]. Given that the loose plant architecture of RIL-D was characterized by a relatively large tiller angle under NSD conditions (Figure 1e–h), we hypothesized that *TAC1* (ORF3) was the candidate locus. To test this possibility, we first sequenced the 5354 bp *TAC1* genomic region (including the 2244 bp predicted promoter) from RIL-D and RIL-C, and found 11 single nucleotide polymorphisms (SNPs) in the promoter region, eight SNPs in the introns, one SNP in the exon 3, and one InDel in the intron 1 (Figure 2e). In particular, SNP20 (Figure 2g), which was located at the splicing site in the fourth intron in 3′-UTR of *tac1* in RIL-C, was the previously described functional SNP (FNP) that leads to abnormal splicing of the 3′-UTR of the *tac1* allele, and thus results in its decreased expression level [5,6]. To determine whether the SNPs in the promoter region contribute to differences in the promoter function, we transformed the plasmids *TAC1^pro^::Luciferase* (*LUC*) and *tac1^pro^::LUC* into rice protoplasts, and used the Dual-Luciferase^®^ Reporter Assay System to compare promoter activities of *TAC1* and *tac1* in RIL-D and RIL-C, respectively. The results showed no significant difference between alleles (Figure 2f), suggesting that these 11 SNPs in the promoter region may not affect the transcriptional activities of the two alleles. In addition, other mutations occurred in introns, such as SNP12 and SNP14-19, or produced a synonymous mutation (ACG to ACA), such as SNP13 (Figure 2e). Therefore, we speculated that the SNP20 (FNP) between *TAC1* and *tac1* could lead to a transition from the dynamic plant architecture observed in RIL-D to the relatively compact plant architecture observed in RIL-C.

### 2.3. TAC1 Is the Target Gene Responsible for the Dynamic Plant Architecture of RIL-D

**Figure 3:**
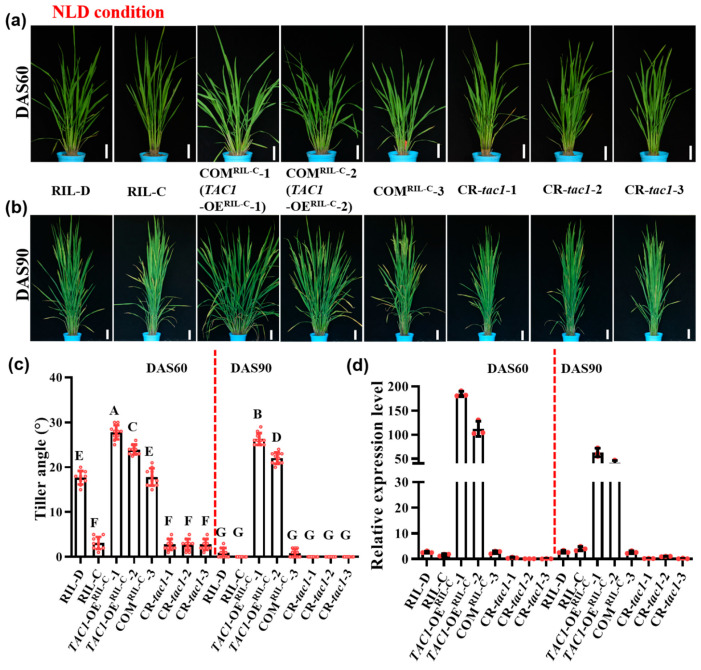
Plant architecture and *TAC1* expression of RIL-D, RIL-C, COM^RIL-C^, and CR-*tac1* lines under natural long-day (NLD) conditions. (**a**,**b**) Plant architecture of RIL-D, RIL-C, COM^RIL-C^-1 (*TAC1*-OE^RIL-C^-1), COM^RIL-C^-2 (*TAC1*-OE^RIL-C^-2), COM^RIL-C^-3, and CR-*tac1*-1/2/3 at the tillering stage (DAS60) (**a**) and heading stage (DAS90) (**b**). (**c**) Multiple comparisons of tiller angle of RIL-D, RIL-C, COM^RIL-C^-1 (*TAC1*-OE^RIL-C^-1), COM^RIL-C^-2 (*TAC1*-OE^RIL-C^-2), COM^RIL-C^-3, and CR-*tac1*-1/2/3. (**d**) *TAC1* expression of RIL-D, RIL-C, COM^RIL-C^-1 (*TAC1*-OE^RIL-C^-1), COM^RIL-C^-2 (*TAC1*-OE^RIL-C^-2), COM^RIL-C^-3, and CR-*tac1*-1/2/3 in the tiller base. Different letters indicate statistical difference at *p* < 0.01 using Duncan’s test. Data are shown as mean ± SD (*n* = 10 for tiller angle measurements, *n* = 3 for expression detection). Bar = 10 cm.

**Figure 4:**
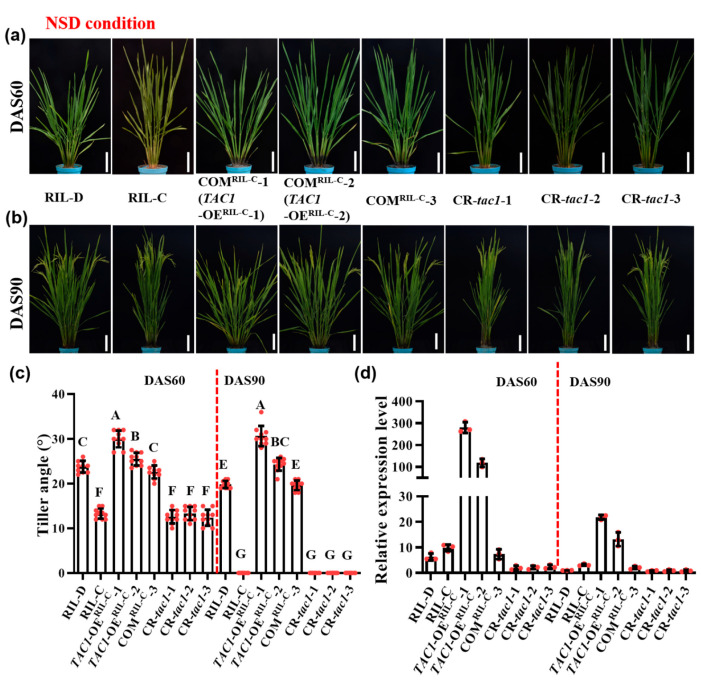
Plant architecture and the *TAC1* expression of RIL-D, RIL-C, COM^RIL-C^, and CR-*tac1* lines under natural short-day (NSD) conditions. (**a**,**b**) Plant architecture of RIL-D, RIL-C, COM^RIL-C^-1 (*TAC1*-OE^RIL-C^-1), COM^RIL-C^-2 (*TAC1*-OE^RIL-C^-2), COM^RIL-C^-3, and CR-*tac1*-1/2/3 at the tillering stage (DAS60) (**a**) and heading stage (DAS90) (**b**). (**c**) Multiple comparisons of tiller angle of RIL-D, RIL-C, COM^RIL-C^-1 (*TAC1*-OE^RIL-C^-1), COM^RIL-C^-2 (*TAC1*-OE^RIL-C^-2), COM^RIL-C^-3, and CR-*tac1*-1/2/3. (**d**) The *TAC1* expression of RIL-D, RIL-C, COM^RIL-C^-1 (*TAC1*-OE^RIL-C^-1), COM^RIL-C^-2 (*TAC1*-OE^RIL-C^-2), COM^RIL-C^-3, and CR-*tac1*-1/2/3 in the tiller base. Different letters indicate statistical difference at *p* < 0.01 using Duncan’s test. Data are shown as mean ± SD (*n* = 10 for tiller angle measurements, *n* = 3 for expression detection). Bar = 10 cm.

To test whether the *TAC1* gene is responsible for the dynamic plant architecture of RIL-D, we generated the complementation (COM) lines COM^RIL-C^ by introducing the plasmid *TAC1^pro^::TAC1* harboring the 6765 bp genomic DNA sequence (including a 2290 bp region upstream of the start codon, a 1280 bp coding region, and a 3195 bp region downstream of the stop codon; Figure S6a) of the *TAC1* gene from RIL-D into the RIL-C background. Moreover, we also generated the *TAC1* gene knockout lines (CR-*tac1*-1/2/3) on the RIL-D background using the CRISPR/Cas9 (CR) system (Figure S6b). Under NLD and NSD conditions, a complementary line (COM^RIL-C^-3) displayed the same plant architecture as that of RIL-D (Figure 3a–c, Figure 4a–c and Figure S7c,g). A previous study demonstrated that *TAC1* is primarily expressed in the tiller base [5]. To understand whether *TAC1* expression is the same in these lines, we detected *TAC1* expression in the tiller base using real-time quantitative PCR (qRT-PCR). As expected, COM^RIL-C^-3 showed similar *TAC1* expression levels to that of RIL-D in the tiller base (Figure 3d and Figure 4d). In addition, *TAC1* knockout lines (the *TAC1* frameshift mutants, CR-*tac1*-1/2/3 (Figure S6b)) with extremely low *TAC1* expression had a compact plant architecture similar to that of RIL-C (Figure 3, Figure 4 and Figure S7d,h). Similar results were also obtained under NSD conditions in the winter season of the Fuyang greenhouse (Figure S1e,f). Furthermore, we also detected the *TAC1* and *tac1* expression levels in RIL-C and RIL-D at different development stages under NLD conditions, and the results show that *TAC1* expression reached the highest levels at DAS50 and had the greatest difference in *tac1* expression, whereas it was lowest at DAS90 and showed no difference in *tac1* expression (Figure S8), which was consistent of the dynamic plant architecture in RIL-D and the compact plant architecture in RIL-C. These results demonstrate that *TAC1* is responsible for the dynamic plant architecture of RIL-D.

### 2.4. Transgenic Lines with High TAC1 Expression Displayed Looser Plant Architecture

In the COM^RIL-C^ lines, we found that the COM^RIL-C^-1 and COM^RIL-C^-2 lines exhibited different and looser plant architecture than those of RIL-D and COM^RIL-C^-3. Under NLD conditions, their respective tiller angles could reach ~27.8° and ~24.0° at DAS60 (tillering stage), and ~26.3° and ~22.1° at DAS90 (heading stage), corresponding to ~17.5° at DAS60 and ~0° at DAS90 in the RIL-D and COM^RIL-C^-3 lines (Figure 3a–c). At the maturation stage, they showed a looser plant architecture with a larger tiller angle and a more curved stem (Figure S7a,b). Consistent with the looser plant architecture, the expression levels of the *TAC1* gene in these two lines significantly increased (Figure 3d). We then renamed COM^RIL-C^-1 and COM^RIL-C^-2 as the *TAC1* overexpression (OE) lines, *TAC1*-OE^RIL-C^-1 and *TAC1*-OE^RIL-C^-2. Similarly, under NSD conditions, the COM^RIL-C^-1 (*TAC1*-OE^RIL-C^-1) and COM^RIL-C^-2 (*TAC1*-OE^RIL-C^-2) lines also displayed a looser plant architecture than those of RIL-D and COM^RIL-C^-3 (Figure 4a–c and Figure S7e,f), and their respective tiller angles were ~30.0° and ~25.5° at DAS60, and ~30.7° and ~24.3° at DAS90, corresponding to ~23.8° at DAS60 and ~19.8° at DAS90 in the RIL-D and COM^RIL-C^-3 lines (Figure 4c). Consistently, the expression levels of the *TAC1* gene in these two lines were still markedly increased (Figure 4d).

### 2.5. Complementary Lines in the Nipponbare (NPB) Background Show Different Degrees of Looseness in Plant Architecture

**Figure 5:**
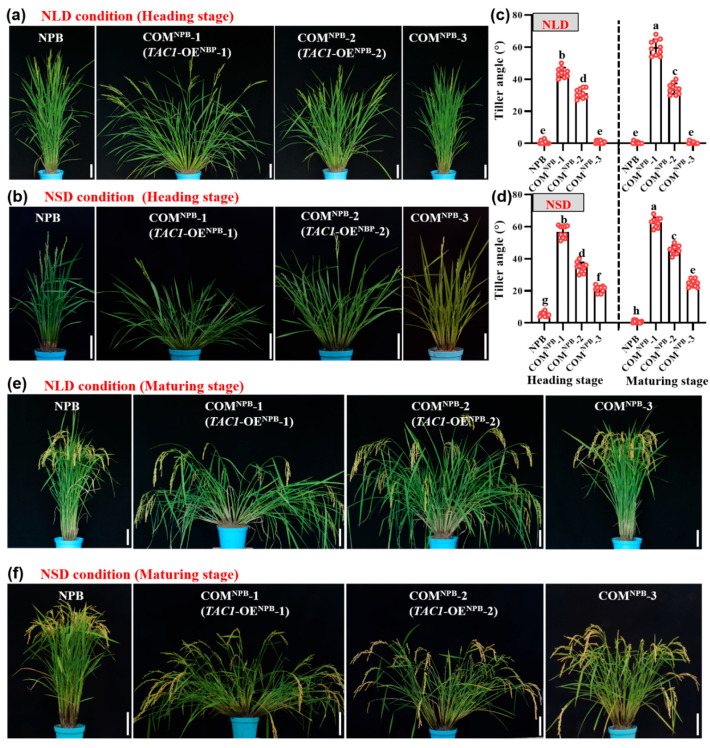
Plant architecture of NPB and COM^NPB^-1/2/3 under natural long-day (NLD) and short-day (NSD) conditions. (**a**,**b**) Plants of NPB (containing the *tac1* allele) and COM^NPB^-1/2/3 at the heading stage under NLD (**a**) and NSD (**b**) conditions. (**c**,**d**) Multiple comparisons of tiller angle of NPB and COM^NPB^-1/2/3 at the heading stage and maturing stage under NLD and NSD conditions. Different letters indicate statistical difference at *p* < 0.05 using Duncan’s test. Data are shown as mean ± SD (*n* = 10). (**e**,**f**) Plants of NPB and COM^NPB^-1/2/3 at the maturing stage under NLD (**e**) and NSD (**f**) conditions. Bar = 10 cm.

To further confirm *TAC1* function in the regulation of plant architecture, we also introduced the *TAC1^pro^::TAC1* into the NPB (containing the *tac1* allele [5]) background to generate the COM^NPB^ lines, in which a similar phenomenon to that of the COM^RIL-C^ lines was found (Figure 5). Notably, the respective tiller angles of COM^NPB^-1 (renamed *TAC1*-OE^NPB^-1) and COM^NPB^-2 (renamed *TAC1*-OE^NPB^-2) reached ~44.2° and ~31.2° at the tillering stage, and ~59.9° and ~34.1° at the heading stage, under NLD conditions (Figure 5c), corresponding to ~27.8° at the tillering stage and ~26.3° at the heading stage in the *TAC1*-OE^RIL-C^-1 line under NLD conditions (Figure 3c). Under NSD conditions, the respective tiller angles of the *TAC1*-OE^NPB^-1 and *TAC1*-OE^NPB^-2 lines appeared to be ~56.8° and ~34.6° at the tillering stage, and ~62.7° and ~45.5° at the heading stage (Figure 5d), corresponding to ~30.0° and ~30.7° in the *TAC1*-OE^RIL-C^-1 line under NSD conditions (Figure 4c). Altogether, the COM^NPB^ lines showed different degrees of looseness in plant architecture, and the *TAC1*-OE^NPB^-1 and *TAC1*-OE^NPB^-2 lines had a looser plant architecture than *TAC1*-OE^RIL-C^-1, indicating that *TAC1* has a greater effect on plant architecture in NPB than in RIL-C.

### 2.6. The Expression of Tiller-Angle-Related Genes Did Not Change between NPB and the TAC1-OE^NPB^-1 Line

**Figure 6:**
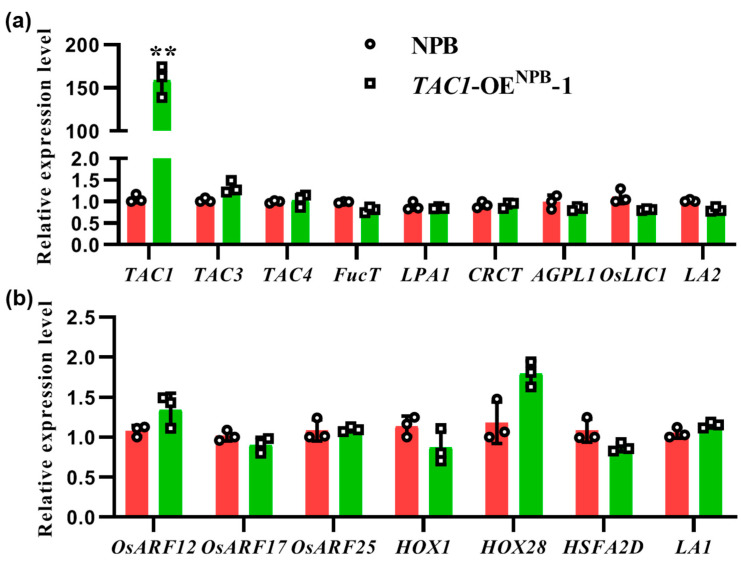
Expression analysis of *TAC1* and tiller-angle-related genes between NPB and the *TAC1*-OE^NPB^-1 line at DAS50 (tillering stage) under natural long-day conditions. (**a**) Expression analysis of *TAC1* and tiller-angle-related genes. (**b**) Expression analysis of tiller-angle-related genes involved in the core regulatory pathway mediated by *LA1*-dependent asymmetric distribution of auxin. All data are shown as mean ± SD (*n* = 3). Two-tail Student’s *t*-test was used for statistical analysis (** *p* < 0.01).

Considering that *TAC1* controls dynamic plant architecture in RIL-D, including the dynamic changes in tiller angle, we assessed whether *TAC1* affects the expression of genes related to tiller angle. To test this possibility, we performed qRT-PCR analysis to detect the expression of the *TAC1* gene and tiller-angle-related genes in NPB and the *TAC1*-OE^NPB^-1 line at the tillering stage (DAS50) under NLD conditions. Regarding the tiller-angle-related genes, we selected 15 genes for qRT-PCR analysis, including *TAC3* [35], *TAC4* [24], *α1,3-fucosyltransferase* (*FucT*) [36], *LPA1* [29,30], *CO2-Responsive CONSTANS, CONSTANS-Like, and Time of Chlorophyll a/b Binding Protein Expression 1* (*CRCT*) [37], *large subunit of ADP-glucose pyrophosphorylase* (*OsAGPL1*) [17], *TILLER ANGLE INCREASED CONTROLLER 1* (*OsLIC1*) [38,39], and *LA2* [22], as well as *Oryza sativa Auxin Response Factor 12/17/25* (*OsARF12/17/25*), *HOX1/28*, *HSFA2D*, and *LA1*, which are involved in the core regulatory pathway mediated by *LA1*-dependent asymmetric auxin distribution [17,18,40]. As a result, the expression of *TAC1* significantly increased in the *TAC1*-OE^NPB^-1 line (Figure 6a), while the expression of all the tested tiller-angle-related genes showed no differences between NPB and the *TAC1*-OE^NPB^-1 line (Figure 6a,b).

## 3. Discussion

### 3.1. TAC1 Is Responsible for the Dynamic Changes in Plant Architecture in Rice

Plants must constantly adjust their architecture to adapt to a changing natural environment. Therefore, a better understanding of the molecular mechanisms underlying these dynamic adjustments can improve crop adaptability to both internal and external conditions. To date, the related factors or genes regulating these dynamic changes in plant architecture have not yet been reported. In this study, RIL-D had a dynamic plant architecture characterized by ‘loose^tiller angle^ (tillering stage)–compact (heading stage)–loose^curved stem^ (maturing stage)’ under NLD conditions, and ‘loose^tiller angle^ (tillering and heading stages)–loose^tiller angle and curved stem^ (maturing stage)’ under NSD conditions, whereas RIL-C was always characterized by a relatively compact plant architecture under both NLD and NSD conditions (Figure 1). Further experiments, including a rice 8K chip test, association analysis, map-based cloning, and gene sequencing, revealed that *TAC1* is the candidate gene for the dynamic plant architecture in RIL-D (Figure 2). This is supported by the fact that *TAC1* is a major QTL controlling rice tiller angle [5], and that RIL-D displays dynamic changes in tiller angle (Figure 1d,h). Sequencing analysis of *TAC1* and a promoter activity assay showed that 11 SNPs in the promoter of *TAC1* in RIL-D and RIL-C did not affect the transcriptional activities of *TAC1* and *tac1* (Figure 2e,f), and the other mutations occurred in introns, or produced a synonymous mutation (Figure 2e). These results suggest that the SNP20, the same mutant site as the *TAC1* FNP identified in *tac1* of IL55 (showing a compact plant architecture with erect tillers similar to that of RIL-C) [5], probably causes the compact plant architecture in RIL-C plants. Subsequently, complementary tests on the RIL-C background and the NPB background demonstrated that *TAC1* is the target gene responsible for the dynamic changes in plant architecture observed in RIL-D (Figure 3, Figure 4 and Figure 5, Figures S1 and S7). Furthermore, *TAC1* frameshift mutants (Figure S6b) in the RIL-D background showed a compact plant architecture, and extremely decreased *TAC1* expression level (Figure 3, Figure 4 and Figure S1), suggesting that *TAC1* does indeed control the dynamic plant architecture. Notably, *TAC1* regulates tiller angle in the *indica* variety IR24 [5], while in RIL-D, *TAC1* controls tiller angle and also modulates dynamic changes in tiller angle and the stem throughout growth. Therefore, *TAC1* is a gene modulating dynamic changes in plant architecture in rice.

### 3.2. TAC1 Positively Regulates Loose Plant Architecture in Rice

In rice, transgenic plants overexpressing *TAC1* have a larger tiller angle, while transgenic plants suppressing *TAC1* expression via RNA interference (RNAi) have a more compact plant architecture in comparison with their corresponding control plants [5]. In maize, a nucleotide mutation in 5′-UTR of *ZmTAC1* decreased its expression level, resulting in a compact plant architecture with a smaller leaf angle [7]. In a peach cultivar, ‘New Jersey Pillar’, SNPs in introns and 3′-UTR in *PpeTAC1* lead to an undetectable transcript and upright growth habit. Similarly, in Arabidopsis, a T-DNA inserted within intron 4 of *AtTAC1* causes its transcript to be undetectable, and the lateral axillary branch angles were found to be significantly narrower in this T-DNA mutant line than in the wild-type [8]. In this study, the complementary lines *TAC1*-OE^RIL-C^-1 and *TAC1*-OE^RIL-C^-2 with high *TAC1* expression levels showed looser plant architecture (including a larger tiller angle and more curved stem) than those of the RIL-D and COM^RIL-C^-3 lines (Figure 3, Figure 4 and Figure S7). Similar results were also obtained in the *TAC1*-OE^NPB^-1 and *TAC1*-OE^NPB^-2 lines in the NPB background (Figure 5). Moreover, *TAC1* has a greater effect on plant architecture in NPB than in RIL-C, which was logically based on the phenotypes (Figure 3, Figure 4 and Figure 5). One possible explanation for this is the difference in genetic background between these two lines, where NPB is a *japonica* rice, while RIL-C has both *indica* and *japonica* genetic backgrounds; however, the connections between *TAC1* and rice genetic background warrant further investigation. Along with previous results, our findings suggest that *TAC1* and its homologs have conserved functions that positively regulate branch angle and/or loose plant architecture in plants. The *tac1* allele containing the FNP (a single mutation in the splicing site of intron 4 in 3′-UTR) shows a reduced expression level due to abnormal splicing in 3′-UTR, which results in a compact plant architecture with a tiller angle close to zero in IL55 [5]. Unlike the case of the *tac1* allele in IL55, the expression levels of the *tac1* allele in RIL-C were not always lower than those of the *TAC1* allele in RIL-D, as shown by the greatest difference in *tac1* expression at DAS50, and no difference at DAS90 (Figure S8), which is consistent with the plant architecture in RIL-D and RIL-C under NLD conditions. To date, the biochemical functions of *TAC1* regulating plant architecture remain largely unclear, and the preliminary results of our study show that *TAC1* overexpression does not affect the expression of all the tested tiller-angle-related genes (Figure 6). Therefore, *TAC1* may not be involved in the mechanisms known to regulate plant architecture. Based on our findings, however, we conclude that *TAC1* positively regulates loose plant architecture in rice.

### 3.3. TAC1 Modulates Different Plant Architecture under NLD and NSD Conditions, Which May Be Related to Light Signals

Light is an essential environmental cue for plant growth and development since it is both an energy source and a developmental signal [41]. Higher plants have evolved complete and sophisticated mechanisms to utilize light energy and light signals. These plants have at least five classes of photoreceptors [42], through which they can perceive light signals and transmit them to downstream mechanisms, such as the central oscillator of the circadian clock [43]. Output signals are then generated to regulate downstream of multiple physiological processes, and these signals impact plant growth and development [44,45,46]. Many genes involved in this process have been identified as key regulators controlling plant architecture. For example, in rice, *Grain Number, Plant Height, and Heading Date7/8* (*Ghd7/8*) encode CO, CO-LIKE, and TIMING OF CAB1 (CCT) domain proteins and the HAP3 subunit of the heme activator protein (HAP) complex, respectively. Their functions and expression are regulated by photoperiod, and can therefore delay heading and increase plant height and panicle size under long-day conditions [47,48,49]. In this study, RIL-D showed a dynamic plant architecture, with dynamic changes in the tiller angle before the heading stage, and a tiller angle close to zero after the heading stage, under NLD conditions (Figure 1a–d). However, it retained a loose plant architecture with a relatively larger tiller angle throughout its growth under NSD conditions (Figure 1e–h). *TAC1* encodes an expressed protein, which belongs to the IGT gene family, that also includes *LAZY* and *DEEPER ROOTING (DRO)* genes [8,50]. In Arabidopsis, *LAZY1*, *LAZY6*, *DRO1*, *DRO2*, and *AtTAC1* are involved in the circadian clock, and are collectively required for light-mediated branch angle orientation [50,51]. For example, light promotes *AtTAC1* expression, while dark inhibits its expression, which leads to narrower lateral branch angles in response to growth in continuous dark versus light [51]. These results show that many members from the IGT gene family can respond to light signals. Our results also show that RIL-D displayed different plant architecture under NLD and NSD conditions, meaning that *TAC1* in rice is most likely regulated by light signals and/or day length. Further experiments are needed to test whether *TAC1* responds to light signals in rice. Based on previous research and our current results, *TAC1* likely regulates plant architecture differently under NSD and NLD conditions through its responses to light signals.

## 4. Materials and Methods

### 4.1. Plant Materials and Growth Conditions

RIL (recombinant inbred line)-D (the RIL with dynamic plant architecture) and RIL-C (the RIL with compact plant architecture) were selected from a population of F_6:7_ RILs derived from single-seed descents from a cross between *indica* variety ZH8015 and *japonica* variety 02428. The NPB contains the *tac1* allele [5]. All plants were cultivated in the paddy field at China National Rice Research Institute (CNRRI) under natural long-day (NLD) conditions in Fuyang (Zhejiang province, 119°95′ E, 30°05′ N) during the summer season, and under natural short-day (NSD) conditions in Lingshui (Hainan province, 110°02′ E, 18°48′ N) during the winter season. Additionally, rice plants were cultivated in the Fuyang greenhouse (NSD conditions) during the winter season for phenotypic characterization. All plants were transplanted with an interplant spacing of 22 cm × 22 cm.

### 4.2. Measurements of Rice Tiller Angle

Tiller angle is defined as the angle between the main culm and the outermost tillers according to previously described methods [35]. A protractor (HANS.W, Taiwan, China) with a long arm was used to measure rice tiller angle (Figure S9). The tiller angle (α) is equal to half of the measured value (β). Each set of data was obtained with measurements from at least 10 individuals.

### 4.3. Rice 8K Chip Assay

Thirty plants with loose plant architecture and 64 plants with compact plant architecture from different lines (or the segregation lines) of F_6:7_ at DAS110 under NSD conditions were used for the rice 8K chip assay to identify the candidate region. Information on the rice 8K chip is displayed in Figure S3 and Table S2. The rice 8K chip assay was performed by China Golden Marker (Beijing) Biotechnology Co., Ltd. (Beijing, China) according to the methods previously described [52]. After genotyping using the 8K chip, association analysis was performed using a general linear model (GLM) and mixed linear model (MLM) with Tassel 5.0 software (Ithaca, New York, NY, United States).

### 4.4. Map-Based Cloning of TAC1

Three segregated lines (HNPF-270/273/274, one of which is displayed in Figure S2) from the recombinant inbred lines (RILs) of F_6:7_ and a population derived from RHL-2 (Figure 2c) were used as the fine mapping populations. For the fine mapping of *TAC1*, 1400 individuals with compact plant architecture and 592 individuals with loose plant architecture from F_6:7_ at DAS110 under NSD conditions were genotyped using polymorphic InDel markers. Additionally, 740 loose plants from RHL-2 of F_7:8_ were genotyped to further narrow down the candidate interval. The new InDel markers were developed based on the sequence polymorphism between the *japonica* cultivar Nipponbare (https://rapdb.dna.affrc.go.jp/ (accessed on 12 December 2018)) and the *indica* cultivar MH63 (http://rice.hzau.edu.cn/rice/ (accessed on 12 December 2018)). The primers for fine mapping are listed in Table S4, and primers for sequencing analysis of *TAC1* are listed in Table S5.

### 4.5. Generation of Constructs and Rice Transformation

To generate the complementary (COM) lines COM^RIL-C^ (in the RIL-C background) and COM^NPB^ (in the NPB background), a 6765 bp genomic DNA sequence of *TAC1* was amplified from RIL-D with the specific primers, and inserted into the *Hind*III site of the binary vector *pCAMBIA1300* to generate the complementary construct *TAC1^pro^::TAC1* (Figure S6a). This was introduced into the *Agrobacterium tumefaciens* strain EHA105, and then transformed into the RIL-D and NPB, respectively, through *Agrobacterium*-mediated transformation. To generate the gene knockout constructs for the CR-*tac1*-1/2/3 (Figure S6b), the *TAC1* target sequence was designed using the online CRISPR-P tool (http://cbi.hzau.edu.cn/cgi-bin/CRISPR) and was then inserted into the *Aar*I site of *pcas9-sgRNA-AarI* backbone under the control of the *OsU3* promoter. The constructed gene knockout plasmid was introduced into the *Agrobacterium tumefaciens* stain EHA105 and transformed into RIL-D via *Agrobacterium*-mediated transformation. The primers for the generation of constructs are listed in Table S6.

### 4.6. RNA Extraction, cDNA Preparation, and Real-Time Quantitative PCR (qRT-PCR)

Total RNA was extracted using a TIANGEN RNAprep Pure Plant Kit (Tiangen Biotech, Beijing, China) according to the manufacturer’s instructions. First-strand cDNA synthesis and qRT-PCR analysis were performed as previously described [53]. The relative expression levels were normalized to the expression level of the rice *UBQ* gene. The comparative critical threshold (ΔΔCt) method was used to calculate gene expression levels according to the previous description [54]. Three technical replicates for each of the three biological replicates were performed. The gene-specific primers used for qRT-PCR analysis are listed in Table S7.

### 4.7. Rice Protoplast Preparation and Transient Transformation

Rice protoplasts were isolated from 10-day-old seedlings under dark conditions. The seedling stems were transversely cut with a razor, as thin as possible, and then transferred to the 0.6 M mannitol for 10 min to adjust the osmotic pressure. They were then incubated with digestion solution (0.6 M mannitol, 0.195% *w*/*v* 2-(N-morpholino) ethanesulfonic acid (MES, pH 5.7), 1% *w*/*v* Cellulase R10 (Yakult Honsha, Tokyo, Japan), 0.5% *w*/*v* Macerozume R10 (Yakult Honsha, Tokyo, Japan), 0.1% *w*/*v* bovine serum albumin (BSA), 1 mM CaCl_2_, and 5 mM β-mercaptoethanol) for approximately 7–12 h with gentle shaking (28 rpm) at 28 °C. The protoplasts were then washed four times with W5 solution (154 mM NaCl, 125 mM CaCl_2_, 5 mM KCl, 5 mM glucose, and 2 mM MES), resuspended in W5 solution, and subsequently stored at 4 °C for at least 30 min. For transient transformation, 10 μg plasmids (or 15 μg for co-transformation), 100 μL protoplasts, and 110 μL polyethylene glycol (PEG)-CaCl_2_ solution (40% *w*/*v* PEG4000, 0.6 M mannitol, and 100 mM CaCl_2_) were gently mixed and placed at 28 °C in the dark for 15 min. Two volumes of W5 solution were then added to stop the transformation. The transformed protoplasts were collected by soft centrifugation, resuspended with W5 solution, and placed at 28 °C in the dark for at least 24 h.

### 4.8. Promoter Activity Assay in the Rice Protoplasts

A 2222 bp *TAC1* (*tac1*) promoter sequence upstream of the start codon from RIL-D and RIL-C was amplified and inserted into the *BamH*I site of the dual-luciferase vector *pGreenII0800-LUC* (containing the firefly and Renilla luciferase encoding sequence, with the *Renilla luciferase* under the control of the CaMV 35S promoter) to generate the *TAC1^pro^::LUC* and *tac1^pro^::LUC*. This was transiently transformed into rice protoplasts for 24 h at 28 °C in the dark. The firefly luciferase activity (LUC) and Renilla luciferase activity (RLUC) were measured using the Dual-Luciferase^®^ Reporter Assay System (Promega, Madison, WI, USA) according to the manufacturer’s instructions. The relative LUC activity was indicated by the ratio of signal values of LUC to those of RLUC. Each measurement was performed with three independent transformants. Primers for the dual-luciferase vector constructions are listed in Table S6.

### 4.9. Statistical Analysis

A two-tail Student’s *t*-test was used for two-group comparisons. The asterisks represent statistical significances at * *p* < 0.05 and ** *p* < 0.01. ANOVA, followed by Duncan’s test, was performed for multiple comparisons. Different letters indicate statistical differences at *p* < 0.05. All data shown represent mean ± SD.

## 5. Conclusions

RIL-D showed dynamic plant architecture under NLD conditions and loose plant architecture under NSD conditions, while RIL-C displayed a compact plant architecture both under NLD and NSD conditions throughout the growth period. *TAC1* is the target gene that modulates dynamic plant architecture in RIL-D. *TAC1* positively regulates loose plant architecture in rice, and high *TAC1* expression cannot affect the expression of tested tiller-angle-related genes. Altogether, this study demonstrates that the *TAC1* gene is necessary for dynamic changes in rice plant architecture.

## Data Availability

The data presented in this study are available on request from the corresponding author.

## Supplementary Materials

The following supporting information can be downloaded at: https://www.mdpi.com/article/10.3390/ijms23094997/s1.
