# Supplementary material for: Tiller Angle Control 1 Is Essential for the Dynamic Changes in Plant Architecture in Rice
Source: Int J Mol Sci. 2022 Apr 30;23(9):4997. doi: 10.3390/ijms23094997 (PMC9105778; doi:10.3390/ijms23094997)
Supplement: Supplementary file 1 [file ijms-23-04997-s001.zip › 2-Supplementary Materials.pdf]

Supplementary Materials

Article title: *Tiller Angle Control 1* Is Essential for the Dynamic Changes in Plant Architecture in Rice

Authors: Hong Wang, Ranran Tu, Lianping Sun, Dongfei Wang, Zheyan Ruan, Yue Zhang, Zequn Peng, Xingpeng Zhou, Qunen Liu, Weixun Wu, Xiaodeng Zhan, Xihong Shen, Yingxin Zhang, Liyong Cao and Shihua Cheng

The following Supporting Information is available for this article:

Figure S1. Plant architecture of RIL-D, RIL-C, COM^{RIL-C}-1 (*TAC1*-OE^{RIL-C}-1), COM^{RIL-C}-2 (*TAC1*-OE^{RIL-C}-2), COM^{RIL-C}-3, and CR-*tac1*-1/2/3 in the winter season of Fuyang green-house (natural short-day conditions).

Figure S2. A segregated population from $F_{6:7}$ at DAS110 under natural short-day conditions.

Figure S3. SNP markers from the rice 8K chip distributed on 12 chromosomes.

Figure S4. Genetic background detection of 30 loose plants and 64 compact plants from different lines from $F_{6:7}$ at DAS110 under natural short-day condition using the rice 8K chip.

Figure S5. The candidate region detection using GLM and MLM with the Tassel 5.0 software.

Figure S6. Information for generation of the transgenic lines.

Figure S7. Plant architecture of COM^{RIL-C}-1 (*TAC1*-OE^{RIL-C}-1), COM^{RIL-C}-2 (*TAC1*-OE^{RIL-C}-2), COM^{RIL-C}-3, and CR-*tac1*-1 at the late maturing stage (DAS120) under natural long-day (NLD) and short-day (NSD) conditions.

Figure S8. Expression level analysis of *TAC1* and *tac1* in RIL-D and RIL-C at DAS40, DAS50, DAS60, and DAS90 under natural long-day condition.

Figure S9. The tool and method for rice tiller angle measurement.

Table S1. Segregation analysis of the candidate locus.

Table S2. Distribution of the SNP markers from the rice 8K chip on chromosomes.

Table S3. Genotypes of 30 loose plants and 64 compact plants from $F_{6:7}$ at DAS110 under natural short-day conditions detected by the rice 8K chip. (See the separate file 'Supplementary-Table S3.xlsx').

Table S4. Primers used for fine mapping of *TAC1*.

Table S5. Primers used for sequencing analysis of *TAC1*.

Table S6. Primers used for generating constructs.

Table S7. Primers used for qRT-PCR analysis.

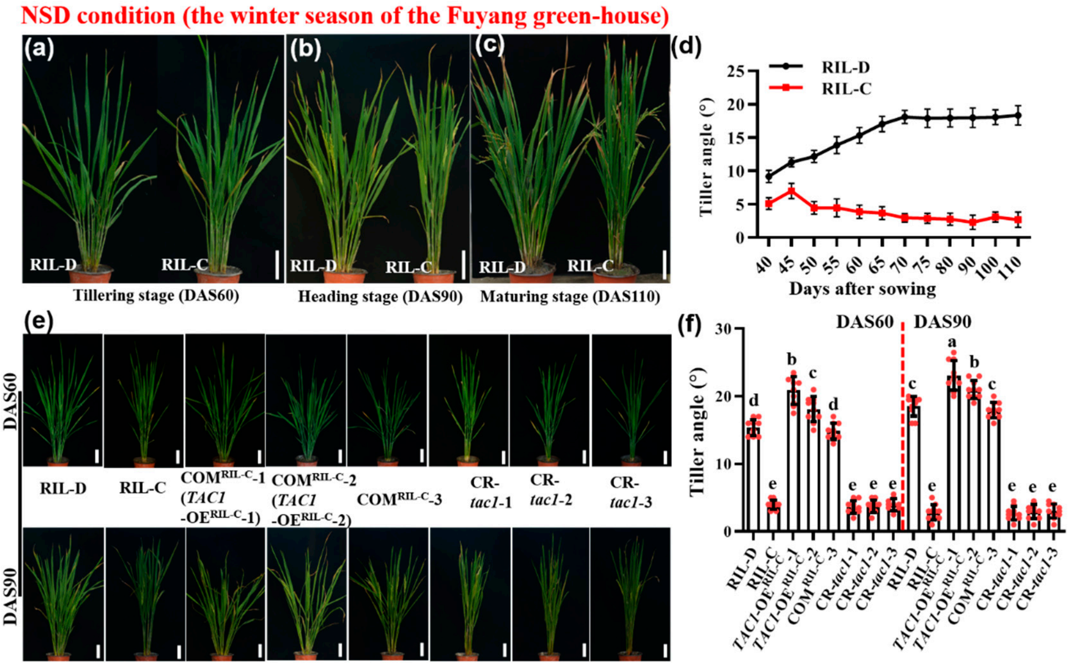

Figure S1. Plant architecture of RIL-D, RIL-C, COM^{RIL-C}-1 (*TAC1*-OE^{RIL-C}-1), COM^{RIL-C}-2 (*TAC1*-OE^{RIL-C}-2), COM^{RIL-C}-3, and CR-*tac1*-1/2/3 in the winter season of Fuyang green-house (natural short-day conditions). (**a**) Plants of RIL-D and RIL-C at tillering stage. (**b**) Plants of RIL-D and RIL-C at heading stage. (**c**) Plants of RIL-D and RIL-C at maturing stage. (**d**) Dynamic tiller angle of RIL-D and RIL-C from DAS40 to DAS110. (**e**) Plants of RIL-D, RIL-C, COM^{RIL-C}-1 (*TAC1*-OE^{RIL-C}-1), COM^{RIL-C}-2 (*TAC1*-OE^{RIL-C}-2), COM^{RIL-C}-3, and CR-*tac1*-1/2/3 at tillering stage (DAS60) and heading stage (DAS90). (**f**) Multiple comparison of tiller angle of RIL-D, RIL-C, COM^{RIL-C}-1 (*TAC1*-OE^{RIL-C}-1), COM^{RIL-C}-2 (*TAC1*-OE^{RIL-C}-2), COM^{RIL-C}-3, and CR-*tac1*-1/2/3 at DAS60 and DAS90. Different letters indicate the statistical difference at $p < 0.05$ by Duncan's test. Data are shown as means ± SDs (n = 10). Bar = 10 cm.

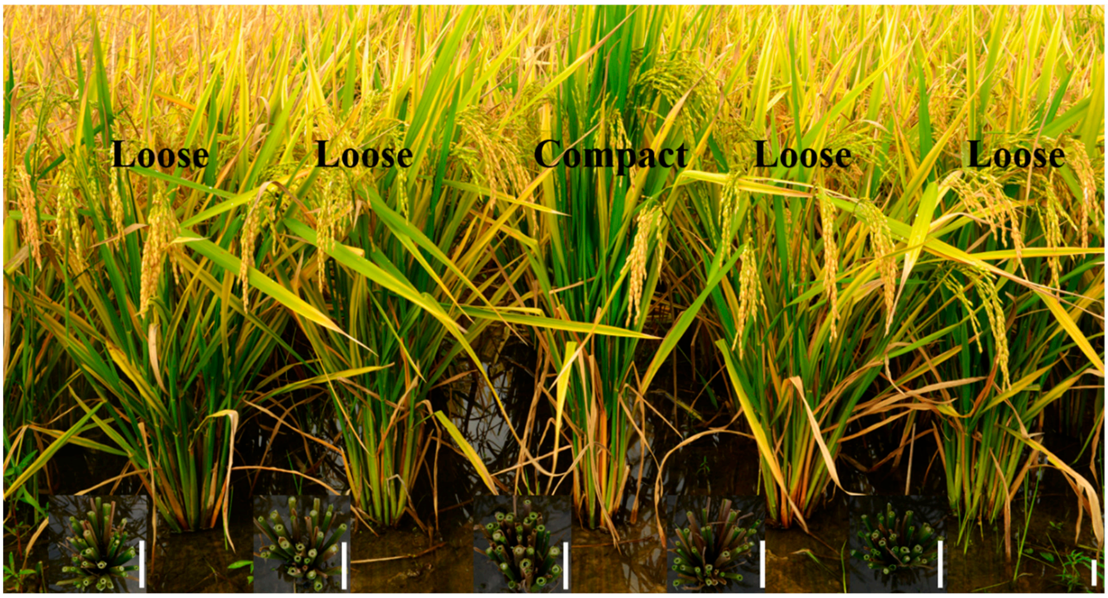

Figure S2. A segregated population from $F_{6:7}$ at DAS110 under natural short-day condition. Bar = 10 cm.

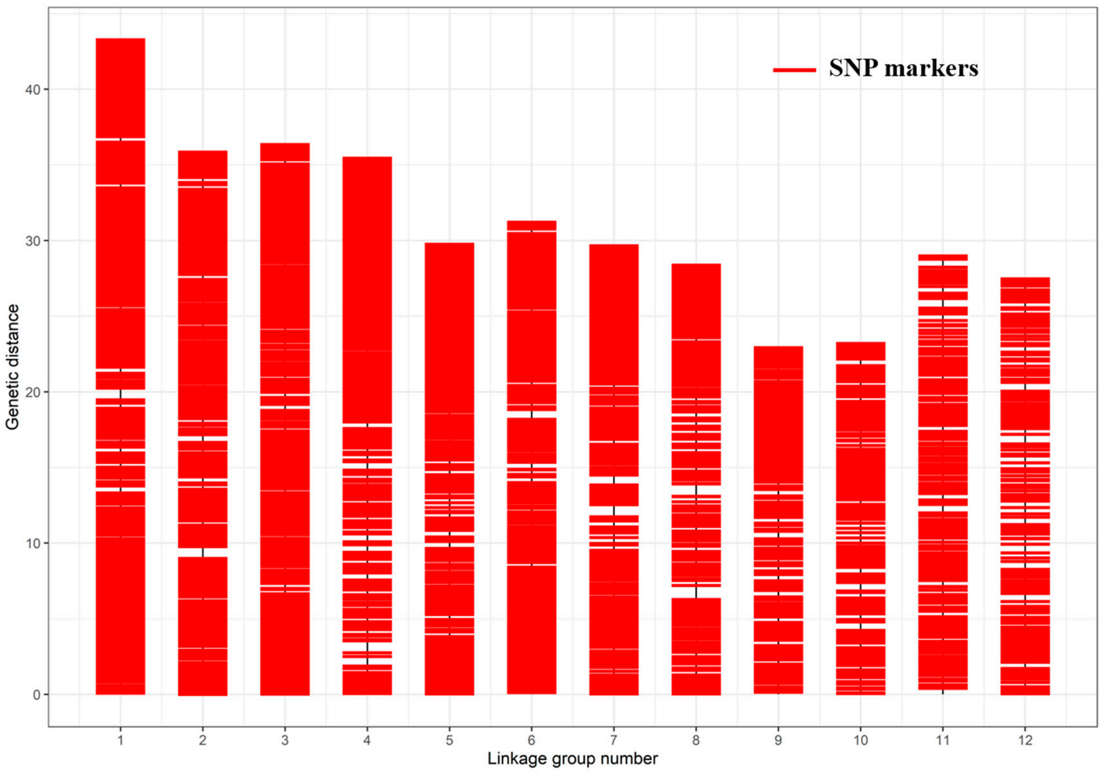

Figure S3. SNP markers from the rice 8K chip distributed on 12 chromosomes. Red lines indicate the SNP markers from the 8K chip.

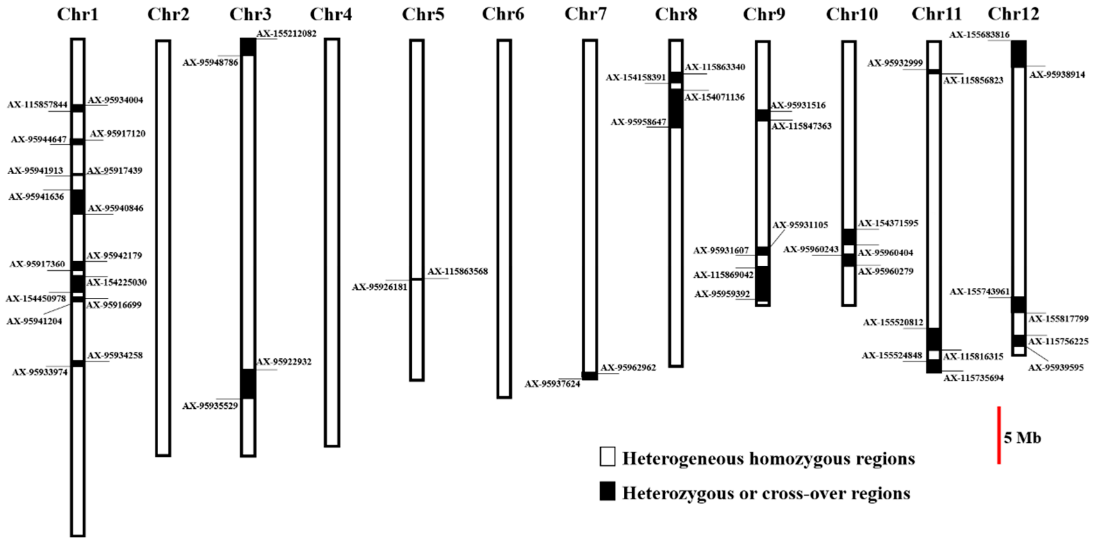

Figure S4. Genetic background detection of 30 loose plants and 64 compact plants from different lines from $F_{6:7}$ at DAS110 under natural short-day condition using the rice 8K chip. AX-number represent the name of SNP marker.

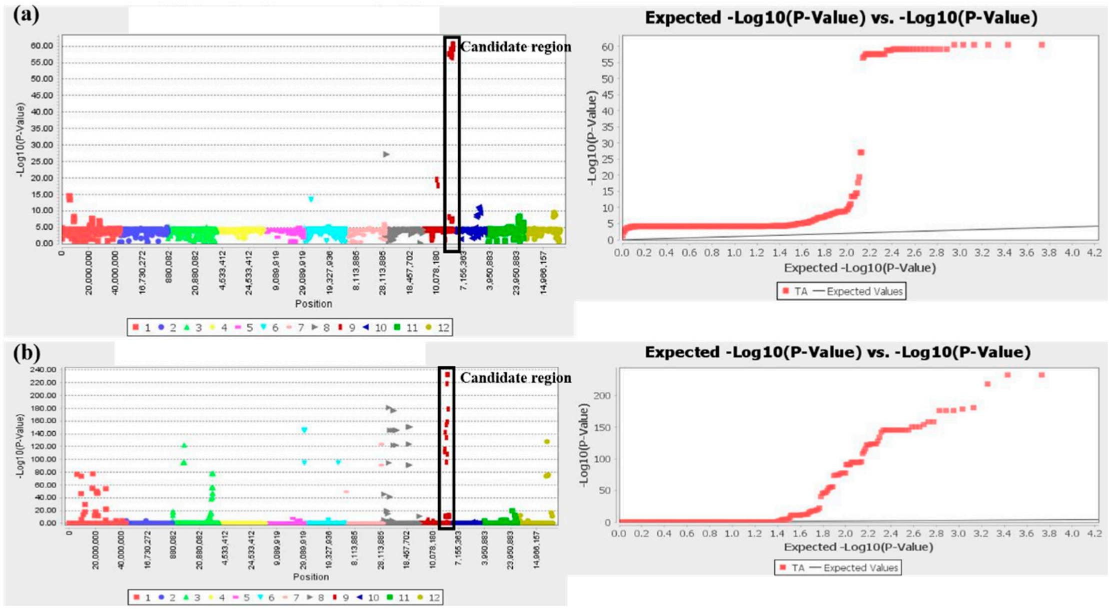

Figure S5. The candidate region detection using GLM and MLM with the Tassel 5.0 software. (**a**) GLM, general linear model. (**b**) MLM, mixed linear model.

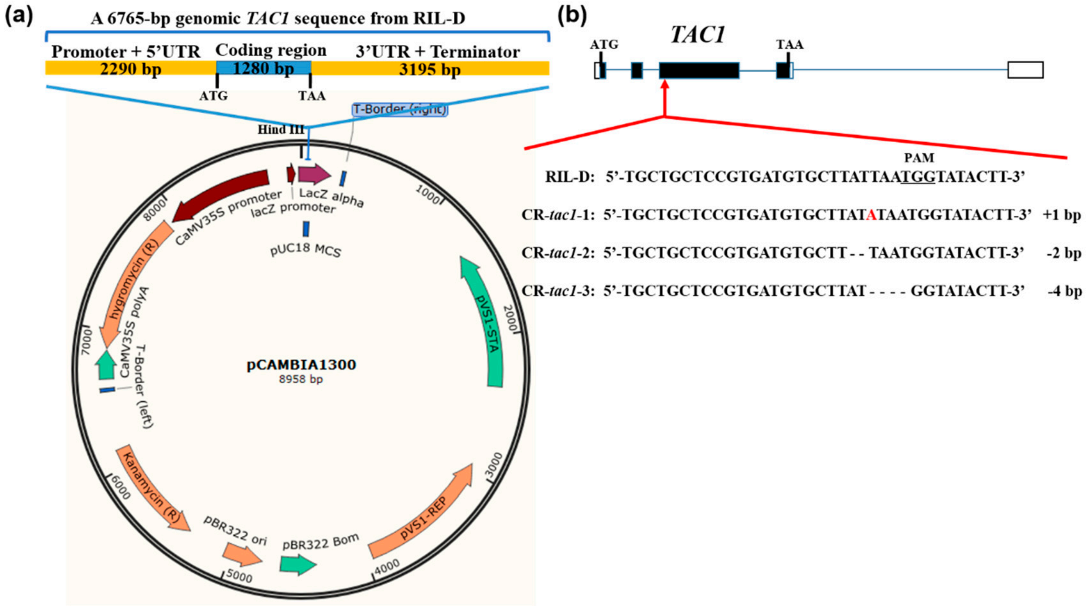

Figure S6. Information for generation of the transgenic lines. (**a**) Construction of the complementation vector *TAC1^pro^::TAC1*. (**b**) Mutation details of the *TAC1* gene knockout lines in the RIL-D background.

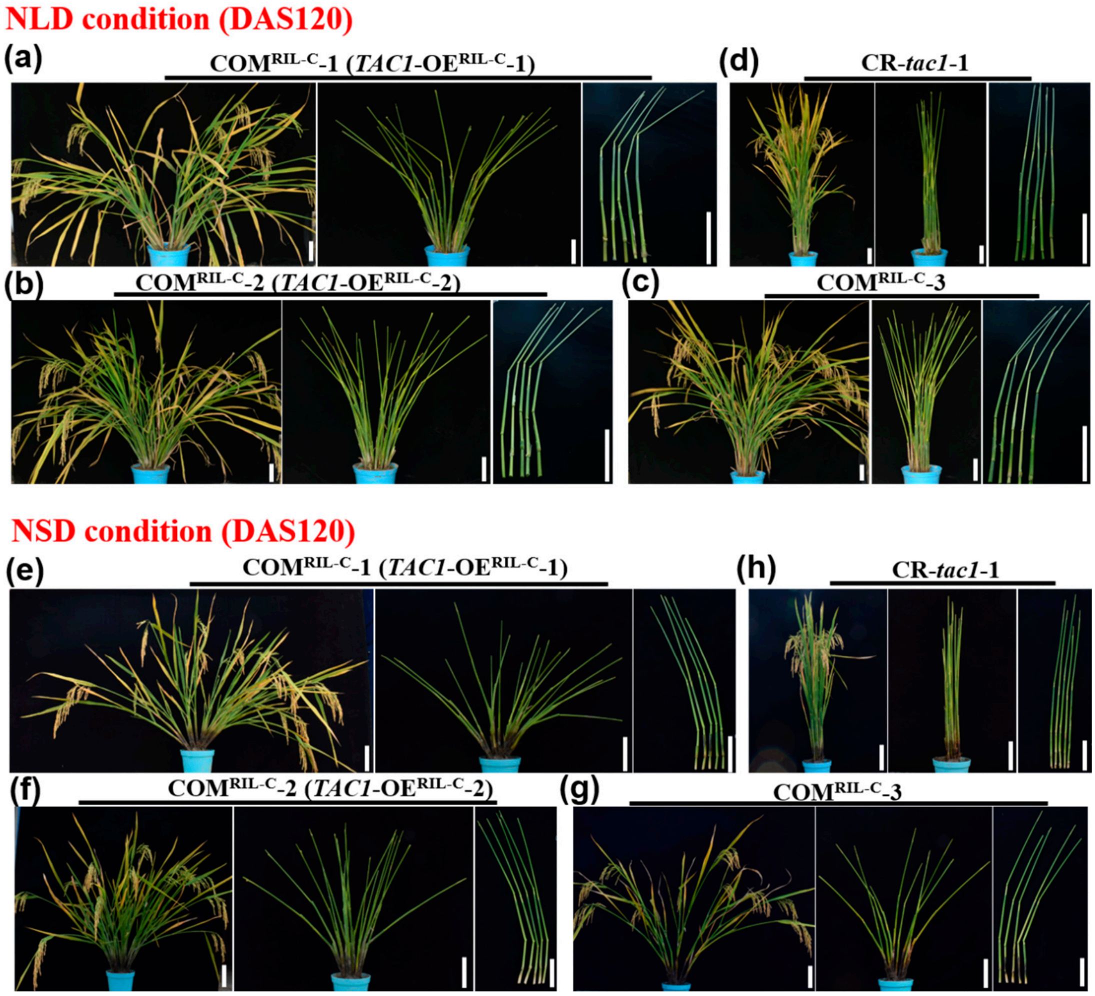

Figure S7. Plant architecture of COM^{RIL-C}-1 (*TAC1*-OE^{RIL-C}-1), COM^{RIL-C}-2 (*TAC1*-OE^{RIL-C}-2), COM^{RIL-C}-3, and CR-*tac1*-1 at the late maturing stage (DAS120) under natural long-day (NLD) and short-day (NSD) conditions. Bar = 10 cm.

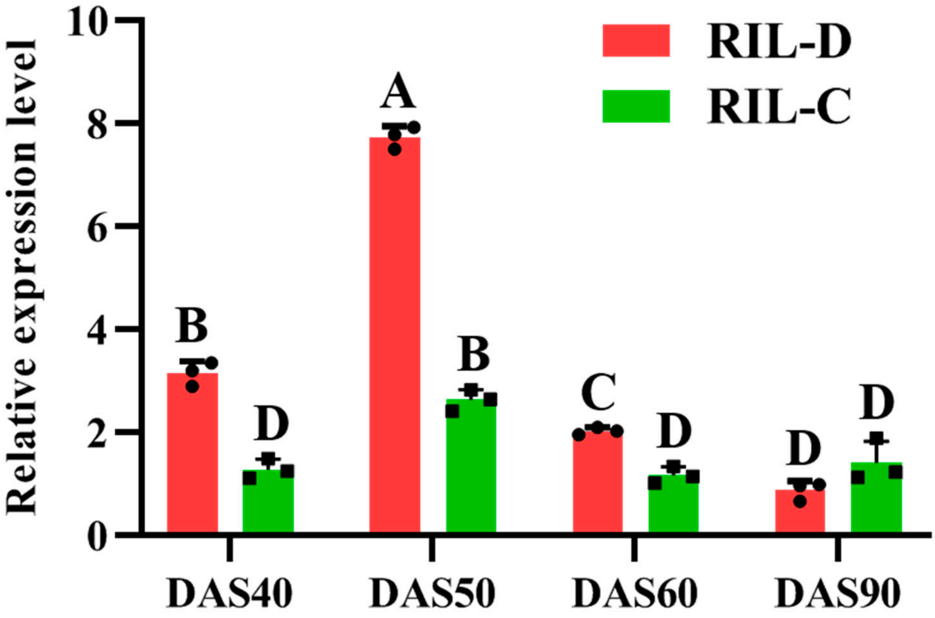

Figure S8. Expression level analysis of *TAC1* and *tac1* in RIL-D and RIL-C at DAS40, DAS50, DAS60, and DAS90 under natural long-day condition. DAS, Days after sowing. Data are shown as means ± SDs (n = 3). Different letters indicate the statistical difference at $p < 0.01$ using Duncan's test.

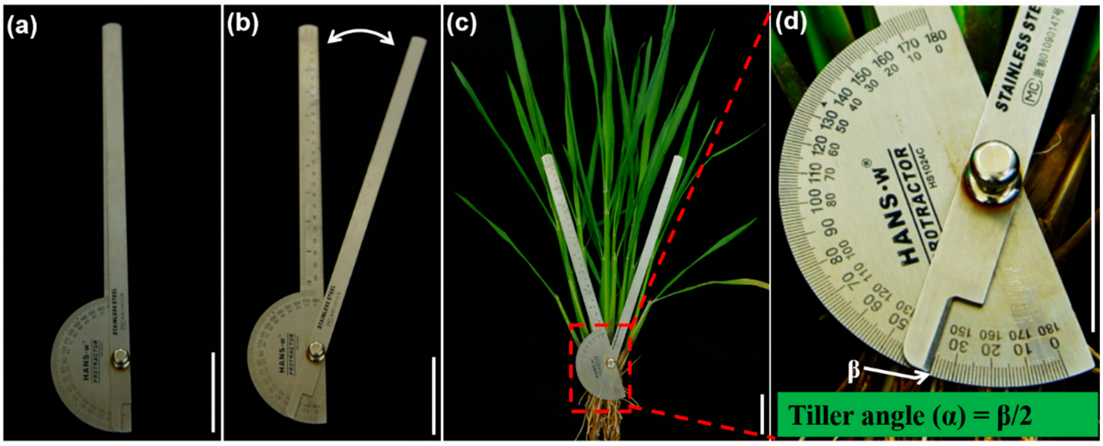

Figure S9. The tool and method for rice tiller angle measurement. (**a**,**b**) A long arm protractor as the tool for tiller angle measurement. (**c**,**d**) The method for tiller angle measurement. The tiller angle (α) is half of the measured value (β). Bar = 5 cm.

Table S1. Segregation analysis of the candidate locus.

RILs	Number of individuals		χ^2	$\chi^2_{3:1}$
	Loose	Compact		
HNPF-270	385	115	1.07	3.84
HNPF-273	382	118	0.52	--
HNPF-274	393	107	3.46	--

Table S2. Distribution of the SNP markers from the rice 8K chip on chromosomes.

Chromosomes	No. of SNP markers	Average distance (bp) between SNP markers
1	1060	40822
2	803	44754
3	776	46925
4	922	38506
5	683	43863
6	851	36720
7	688	43165
8	494	57577
9	429	53563
10	225	103143
11	351	82681
12	237	116168

Table S3. Genotypes of 30 loose plants and 64 compact plants from $F_{6:7}$ at DAS110 under natural short-day conditions detected by the rice 8K chip. (See the separate file 'Supplementary-Table S3.xlsx')

Table S4. Primers used for gene mapping.

Name	Forward primer sequences(5'-3')	Reverse primer sequences(5'-3')
QP-2	GACATTTAATTCCCCCGCC	CATATTCTAATGCCGCGCTA
QP-17	CTGAAACTTTGGAGATTCATCG	TTGGCTTCACCCATCGAAAATA
QP-36	GACTACATTTAGTTTGCTGCC	GTAAAACGGAGGAAGTAGTCAT
SCR-16	CTCACGAGATGAATGGACAA	ATCCCGCGGTTCGATT
SCR-22	ATCACGAAGTTGCTCTGTTG	AGCCTTTGCTTTTAGATCGT
SCR-26	ACATTCACCCTATGAACCTATGA	GTTTCTTGTTGTGGAACTAGC
SC-3	ATAAAACGATCGCCTTTACGG	ATCATACAAGTTATTTACTCCCCC
SC-12	GATGACAAGTTGAGTGACCT	GAGAGAGATGCTAAGTAACGG
SC-16	ACAAATACATGATACACAGAGGTA	GAGTTCGTAACAACAGCATTT

Table S5. Primers used for sequencing analysis of *TAC1*.

Name	Forward primer sequences(5'-3')	Reverse primer sequences(5'-3')
TAC1-1	CGAGCGAATAGGCAGCAATA	AGGGATTAGGAAGCATGGGT
TAC1-2	GTCCACTGGCCTTTTTCAGA	AGAACTGAATAGCACAGGAAAGT
TAC1-3	GAGTGTCAAGCAGCACTTAGA	CAGTGCGCCACATCTTACAC
TAC1-4	TAGCATTATGCAGTGACAGGG	GAGGCTAAACAGGACACACC
TAC1-5	GAAGTGGTCGTTCCTGGATG	GATCTGAGCTACTGTCTGGC
TAC1-6	ATTGAACACCTGGTAGTGCC	CCATCTCTTTGGAAAGCTGGA
TAC1-7	CACTGACAGAACGGATCAGG	ACGTAGGGATGGAGTGTCAA
TAC1-8	GTTCAGTTGAAGGTTCCCTGT	TGTTGAAGCTTTCGGTGTCA
TAC1-9	CCAGTGGGGATGAGCAAAATA	TGATGTTCACTCCCAGGAATTT

Table S6. Primers used for generating constructs.

Constructs	Primer name	Primer sequences(5'-3')
TAC1^{pro}::TAC1	C-*TAC1*-HindIII-F	GCAGGCATGCAAGCTTATCCTTCTTAAGTGCCCTACAC
	C-*TAC1*-HindIII-R	GGCCAGTGCCAAGCTTCGCCCTTACAACCAACACAA
CR	CR-*tac1*-AarI-F	AGATGATCCGTGGCACTCCGTGATGTGCTTATTAAGTTTTAGAGCTATGC
	CR-*tac1*-AarI-R	GCATAGCTCTAAAACTTAATAAGCACATCACGGAGTGCCACGGATCATCT
TAC1^{pro}::LUC	Pro-*TAC1*-BamHI-F	GCAGCCCGGGGGATCCGTACTATTGATGTTCACTCCCAGG
	Pro-*TAC1*-BamHI-R	TAGAACTAGTGGATCCCTCTCTAGAACCAATATGAAACCAG
tac1^{pro}::LUC	Pro-*tac1*-BamHI-F	GCAGCCCGGGGGATCCGTACTATTGATGTTCACTCCCAGG
	Pro-*tac1*-BamHI-R	TAGAACTAGTGGATCCCTCTCTAGAACCAATATGAAACCAG

Table S7. Primers used for qRT-PCR analysis.

Name	Forward primer sequences(5'-3')	Reverse primer sequences(5'-3')
TAC1	AGATGGCTCTAAAGGTGTTCAA	TCTTCCATGGCCTTGTTCTC
TAC3	CTCACATTAACTTCCAGCACCAA	TCGGTGGATGATGAGGAGAG
TAC4	AAGGTCGCAAACAAGCAG	AACTGCCAGGAGCAGAGAG
FucT	GGAGTCTGCTGTGCTTGCTA	ACTGGTATAATGCCTGTCGTTGTG
LPA1	GCGTATGTATGTAAAGCAAG	GAAACGACCTACGAAACTAC
CRCT	TTCTGGGTGCCTCAACTCA	AACGCTGTCTCAAAGTCCAATC
AGPL1	TTGATTCCACATGGCAGAGAAC	GTTGCTGCTGCTACTTCACT
OsLIC1	GGATGATAGCATCTGGTTGAAGGA	CACATGGCTAACGTGCCTCTG
LA2	GAACCAGCAGCCTGTAAGA	AGCCATCCTCTCCTTCATTG
OsARF12	GTTGGGAGGTCGTTGGACATAA	AAGCACATCATTCTCCCTGTCG
OsARF17	TTTACAAATCGGGAACCTATGG	TTTATGCAGGAGACGCTATTCA
OsARF25	TGACATCTCCAGATTCAGCAGC	CGTCTCCACCACGAACCAA
HOX1	AGCACAACACCCTCAATC	GTTCTGGAACCACACCTC
HOX28	CATTGACCACCCTCACAA	GAATCCGCACAAGAACTG
HSFA2D	CAGCAGGCACTTGGCACC	TTCTTGTCACGCTTTAGCCTGT
LA1	GAGATGAACGGCAACAAG	TTCCAGCACCAAGTAGTC
UBQ	GCTCCGTGGCGGTATCAT	CGGCAGTTGACAGCCCTAG
